# Long-term outcomes of busulfan plus melphalan-based versus melphalan 200 mg/m^2^ conditioning regimens for autologous hematopoietic stem cell transplantation in patients with multiple myeloma: a systematic review and meta-analysis

**DOI:** 10.1186/s12935-021-02313-z

**Published:** 2021-11-10

**Authors:** Fei Gao, Mei-Si Lin, Jie-Shu You, Min-Yue Zhang, Long Cheng, Ke Lin, Peng Zhao, Qi-Yan Chen

**Affiliations:** 1 State Key Laboratory of Southwestern Chinese Medicine Resources, Pharmacy School, Chengdu University of Traditional Chinese Medicine, Chengdu, 611130 China; 2 College of Pharmacy, Shenzhen Technology University, Shenzhen, 518118 Guangdong Province China; 3 Division of Hematology, Renji Hospital, School of Medicine, Shanghai Jiaotong University, Shanghai, 200127 China; 4 Sichuan Provincial Acupuncture School, Chengdu, 611731 China; 5 Department of Cardiology, Gongli Hospital, Shanghai, 200135 China; 6 School of Medical Information Engineering, Chengdu University of Traditional Chinese Medicine, Chengdu, 611130 China

**Keywords:** Multiple myeloma, Busulfan, Melphalan, Autologous hematopoietic stem cell transplantation, Meta-analysis

## Abstract

**Background:**

High-dose melphalan (HDMEL, 200 mg/m^2^) is considered as the standard conditioning regimen for autologous hematopoietic stem cell transplantation (auto-HSCT) in multiple myeloma (MM). However, whether the combination of melphalan with busulfan (BUMEL) conditioning outperforms HDMEL remains controversy. Accordingly, a systematic review and meta-analysis was carried out to compare the outcomes of HDMEL and BUMEL-based conditioning regimens in newly diagnosed MM patients having undergone auto-HSCT.

**Methods:**

A systematic literature search was conducted in PubMed, Embase and Cochrane Library database until July 31, 2021, to identify all eligible studies comparing progression-free survival (PFS), overall survival (OS), optimal treatment response after auto-HSCT, duration of stem cell engraftment and incidence of toxic events between patients undergoing BUMEL-based and HDMEL conditioning regimens. Hazard ratio (HR), mean difference (MD) or odds ratio (OR) corresponding to 95% confidence interval (CI) were determined to estimate outcomes applying RevMan 5.4 software. Publication biases were assessed by performing Egger’s test and Begg’s test by Stata 15 software.

**Results:**

Ten studies with a total of 2855 MM patients were covered in the current meta-analysis. The results of this study demonstrated that patients having received BUMEL-based regimen was correlated with longer PFS (HR 0.77; 95% CI 0.67~0.89, P = 0.0002) but similar OS (HR 1.08; 95% CI 0.92~1.26, P = 0.35) compared with those having received HDMEL. The differences of best treatment response after auto-HSCT and duration of neutrophil or platelet engraftment did not have statistical significance between the two groups of patients. With respect to adverse effects, the patients in BUMEL-based group were less frequently subject to gastrointestinal toxicity while the patients in HDMEL group less often experienced mucositis and infection. No significant difference was observed in hepatic toxicity between the two groups of patients.

**Conclusions:**

In the present study, BUMEL-based conditioning was identified as a favorable regimen for a better PFS and equivalent OS as compared with HDMEL, which should be balanced against higher incidences of mucositis and infection. BUMEL-based conditioning is likely to act as an alternative strategy to more effectively improve auto-HSCT outcomes in MM.

**Supplementary Information:**

The online version contains supplementary material available at 10.1186/s12935-021-02313-z.

## Background

Multiple myeloma (MM) refers to a hematological malignancy, as manifested by excessively proliferated monoclonal plasma cells in the bone marrow, thereby causing renal dysfunction, bone marrow failure and bone destruction. Over the past two decades, the incidence of MM has increased globally by 126% whereas the age-standardized mortality rate has been steadily falling [1] due to the utilization of novel agents [e.g., immunomodulatory drugs (IMiDs), the proteasome inhibitors (PIs), monoclonal antibodies, BCL2 inhibitor, etc.] in the treatment of MM [2–4]. Moreover, high-dose chemotherapy followed by autologous hematopoietic stem cell transplantation (auto-HSCT) is recognized as an effective therapy as consolidation after induction treatment for newly diagnosed MM patients aged less than 65 years [5].

Even so, the disease remains incurable, and considerable patients are eventually subject to disease relapse. Several approaches concentrating on different stages of MM treatment have been developed to down-regulate the incidence of disease progression, including improving the induction of chemotherapy prior to auto-HSCT by combining novel agents [2–4], and applying maintenance treatment with IMiDs or PIs following auto-HSCT [6–8]. Furthermore, existing evidence revealed that MM patients might benefit from intensifying pre-transplantation conditioning chemotherapy to mitigate disease relapse and prolong survivals [9].

Thus far, high-dose melphalan (HDMEL, 200 mg/m^2^) has been considered as the universal standard conditioning regimen for auto-HSCT in MM [5]. Currently, ongoing efforts are being made to enhance the efficacy of pre-transplant conditioning chemotherapy. Other alternative conditioning regimens (e.g., increasing the dose of melphalan to 220 mg/m^2^ and total body irradiation followed by melphalan 140 mg/m^2^) did not reveal any convincing superiority over HDMEL 200 mg/m^2^ and was correlated with increased hematologic and nonhematologic toxicities [10, 11]. Among a wide range of regimens, busulfan has achieved substantial efficacy when combined with melphalan as conditioning for MM auto-HSCT [12–15]. A recent randomized controlled trial (RCT) demonstrated the encouraging results, which showed that the combination of busulfan plus melphalan 140 mg/m^2^ (BUMEL) as pre-transplantation conditioning could achieve a significantly prolonged progression-free survival (PFS) compared with HDMEL 200 mg/m² [15]. According to several retrospective analyses, however, no difference of PFS was identified between MM patients having received conditioning of BUMEL and HDMEL [12–14]. Recognizing the controversial of data regarding BUMEL conditioning in MM patients having undergone upfront auto-HSCT, the present systematic review and meta-analysis was conducted to compare the efficacy and safety of BUMEL with those of HDMEL as the conditioning regimen for auto-HSCT in MM.

## Methods

### Identification of relevant studies

The current study was performed according to Preferred Reporting Items for Systematic Reviews and Meta-analysis (PRISMA) reporting guidelines (see Additional file 1: Table S1) [16]. To identify all studies that compared the efficacy with conditioning of BUMEL-based regimen with HDMEL alone in MM patients having undergone auto-HCT, we performed a comprehensive literature search in PubMed, Embase and the Cochrane Library databases up to July 31, 2021. The details of the search strategy are summarized in Additional file 1: Table S2. Furthermore, the references of retrieved studies, meeting abstracts and meta-analyses were screened. Besides, case reports, editorials and review studies were excluded. When a publication overlapped with other publication of the same trial, only the study with more elucidations or the most recent study was included.

### Selection criteria

Two authors (M Lin and J You) were independently assessed all the studies. The studies included in the present meta-analysis should abide by the criteria below: (1) clinical trials, including prospective or retrospective control studies; (2) patients with newly diagnosis MM; (3) patients having undergone auto-HSCT with conditioning of busulfan plus melphalan-based regimen (BUMEL-based group) or high-dose melphalan alone (HDMEL group); (4) providing auto-HSCT related outcomes measurement between the two groups of patients. Exclusion criteria were: (1) relapse and refractory MM patients; (2) patient number less than or equal to ten in any study groups. When the relevant data was not reported in paper, we contacted the author to get the relevant information by e-mail or telephone.

The primary outcome referred to PFS of patients having received BUMEL-based regimen or HDMEL regimen. The secondary objectives were to compare overall survival (OS), best treatment response after auto-HSCT [very good partial response (VGPR) or better], duration of stem cell engraftment and incidence of toxic effects between the two groups of patients.

### Data extraction and quality assessment

Two reviewers (F Gao and M Zhang) independently extracted the data and assessed the quality of included studies. The following information from the respective study was summarized: (1) first author, (2) year of publication, (3) country, (4) induction treatment regimens, (5) conditioning regimens, (6) number of patients in each arm of study, (7) follow-up time, (8) number of patients having received maintenance treatment, and (9) primary and secondary outcomes of current study.

The quality of retrospective studies was assessed by adopting the Methodological Index for Non-Randomized Studies (MINORS) scale [17]. Given the MINORS scale, 12 methodologic criteria were estimated for comparative studies. The respective item was scored 0 (not reported), 1 (reported but inadequate), or 2 (reported and adequate). Additionally, The methodological quality of RCT was assessed by the Cochrane collaboration’s risk of bias tool [18], which comprised of the following six domains: random sequence generation, allocation concealment, blinding of participants and personnel, blinding of outcome assessment, incomplete outcome date and selective reporting. The respective domain was assessed by employing three levels (i.e., “low,” “high,” and “unclear”). Any discrepancy between the two reviewers was resolved by an additional investigator, L Cheng.

### Statistical analysis

Hazard ratio (HR) corresponding to 95% confidence interval (CI) was adopted to assess OS and PFS. If the results of univariate and multivariate analyses were reported in the included studies, the latter will be applied to the meta-analysis. If HRs and 95% CIs were not available from the original study, Kaplan–Meier curves of the included studies were read and re-analyzed by software Engauge digitizer. HRs and 95% CIs were indirectly calculated from Kaplan–Meier curve using Tierney’s methods [19]. Mean difference (MD) corresponding to 95% CI were applied to assess the duration of stem cell engraftment. If mean and standard deviation (SD) were not reported in the original studies, mean and SD were indirectly estimated from samples size, median and range by using Luo’s method [20] and Wan’s [21] method, respectively. Odds ratio (OR) corresponding to 95% CI were calculated to estimate other outcomes. Publication biases were assessed by performing Egger’s test and Begg’s test. The methods of the meta-analysis and publication biases tests were previously elucidated [22–24]. Statistical analysis was conducted by software ReviewManager 5.4 (The Cochrane Collaboration, Oxford, UK) and Stata version 15 (Stata Corp, College Station, Texas, USA). All P-values were both-sided. P-value of < 0.05 was considered to be statistically significant.

## Results

### Characteristics of studies

**Fig. 1:**
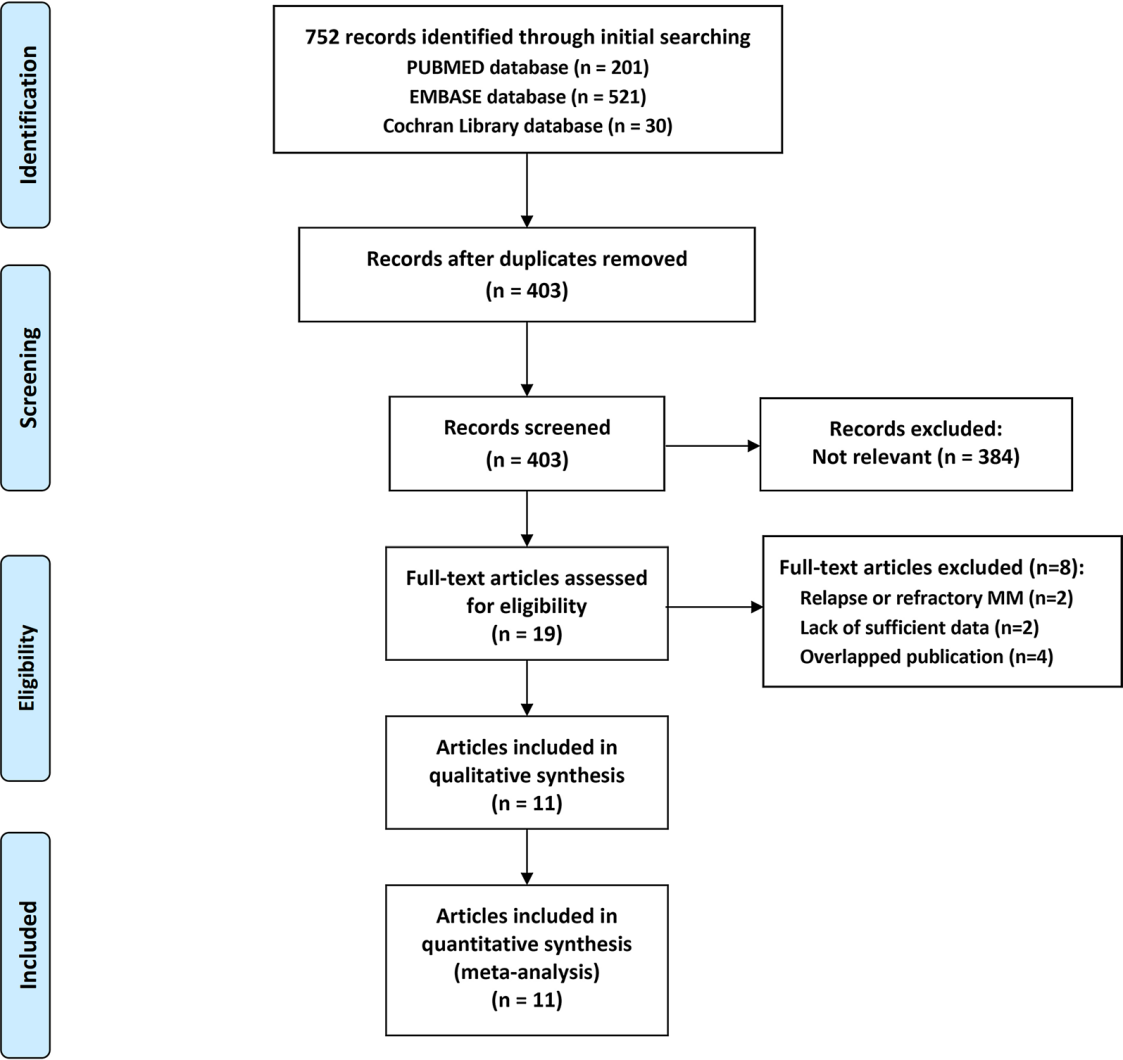
Literature search and selection

Figure 1 presents the flowchart of literature search and selection of evidence. In total, 752 citations were retrieved from the databases and 741 were excluded from the preset meta-analysis by further assessment of the title, abstract or full-text. Lastly, 11 articles [12–15, 25–31] (10 studies comprising 1 RCT and 9 retrospective studies) with a total of 2855 MM patients fulfilled the inclusion criteria and were included in present meta-analysis. The characteristics of the included studies are listed in Table 1. The studies were published from 2002 to 2021. Sample size ranged from 43 to 767. The included studies were conducted in Korea (n = 3), Spain (n = 4), Italy (n =1) and the United States (n = 2). All the patients in the HDMEL group underwent auto-HSCT with conditioning of high-dose melphalan 200 mg/m^2^. Patients in the BUMEL-based group received conditioning regimen of busulfan plus melphalan in nine studies while received busulfan, melphalan plus bortezomib in one study. All the included studies had reliable quality as indicated by final scores of MINORS scale ranged from 16 to 19 for nine retrospective studies [12–14, 25–31] (Additional file 2: Fig. S1) and low risk for each item of Cochrane collaboration’s risk of bias tool for one RCT study [15].

**Table 1:** Baseline characteristics of included studies

Author	Year	Country	Induction treatment	Conditioning regimens	No. patients	Follow-up (m) Median (range)	Maintenance treatment (n)	≥ VGPR pre-ASCT (n)
Bashir [15]	2019	USA	VRD, VCD, KRD, VD, CBAD, RD	Busulfan 32 mg/m^2^, Day-7 to -4, iv; Melphalan 70 mg/m^2^/d, Day-2 to -1	104	22.6 (IQR 15.2–47.1)	87	54
				Melphalan 200 mg/m^2^, Day-2	98	20.2 (IQR 8.8–46.6)	84	56
Blanes M [25, 26]	2019	Spain	VBMCP, VBAD, VAD	Busulfan 3.2 mg/kg/d, Day-5 to -3, iv; Melphalan 140 mg/m^2^, Day-2	51	50	33	–
				Melphalan 100 mg/m^2^/d, Day-3 to -2 or 200 mg/m^2^, Day-2	102	63	65	–
Byun [14]	2018	Korea	–	Combined busulfan (iv) and melphalan	107	mean 37.6 (SD 17.9)	–	–
				Melphalan 200 mg/m^2^	428	mean 37.5 (SD 28.1)	–	–
Galindo R [31]	2006	Spain	-	Busulfan 16 mg/kg, iv; Melphalan 140 mg/m^2^	20	–	–	–
				Melphalan 200 mg/m^2^	23	–	–	–
Hagen P [30]	2020	USA	-	Busulfan 130 mg/m^2^/d, Day-6 to -3, iv; Melphalan 140 mg/m^2^, Day-2; Bortezomib 1.6 mg/m^2^, Day-1	43	86 (5–109)	0	18
				Melphalan 200 mg/m^2^, Day-2	162	85 (1–121)	121	76
Lahuerta [27]	2002	Spain	–	Busulfan 3 mg/kg/d, Day –6 to –3, orally; Melphalan 140 mg/m^2^, Day–2	186	-	80	–
				Melphalan 100 mg/m^2^/d, Day-3 to -2 or 200 mg/m^2^ Day-2	472	-	184	–
Lahuerta JJ [28]	2010	Spain	VBMCP, VBAD	Busulfan 3 mg/kg/d, Day–6 to –3, orally; Melphalan 140 mg/m^2^, Day –2	225	72	–	–
				Melphalan 100 mg/m^2^/d, Day -3 to -2 or 200 mg/m^2^ Day -2	542	47	–	–
Park [12]	2021	Korea	VTD	Busulfan 3.2 mg/kg/d, Day -6 to -4, iv; Melphalan 70 mg/m^2^/d, Day -3 to -2	31	22.4	13	16
				Melphalan 100 mg/m^2^/d, Day-3 to -2	79	34.2	2	53
Ria [29]	2004	Italy	VAD	Busulfan 4 mg/kg/d, Day-7 to -4; Melphalan 100 mg/m^2^, Day-3	14	–	14	–
				Melphalan 200 mg/m^2^, Day-1	16	–	16	–
Song [13]	2020	Korea	TD, CTD	Busulfan 3.2 mg/kg/d, Day -6 to -4, iv; Melphalan 70 mg/m^2^/d, Day-3 to -2.	76	37.3	29	37
				Melphalan 100 mg/m^2^/d, Day-3 to -2	76	50.8	18	43

**Abbreviations: **CBAD: cyclophosphamide-bortezomib-adriamycin-dexamethasone; CTD: cyclophosphamide-thalidomide-dexamethasone; IQR: inter-quartile range; iv: intravenously; KRD: carfilzomib-lenalidomide-dexamethasone; RD: lenalidomide-dexamethasone; VAD, vincristine-adriamycin-dexamethasone; VBAD, vincristine-carmustine-adriamycin-dexamethasone; VBMCP, vincristine-carmustine-melphalan-cyclophosphamide-prednisone; VCD: bortezomib-cyclophosphamide-dexamethasone; VD: bortezomib-dexamethasone; VGPR: very good partial response; VRD: bortezomib-lenalidomide-dexamethasone; VTD: bortezomib-thalidomide-dexamethasone; TD: thalidomide-dexamethasone

### Survival analysis

**Fig. 2:**
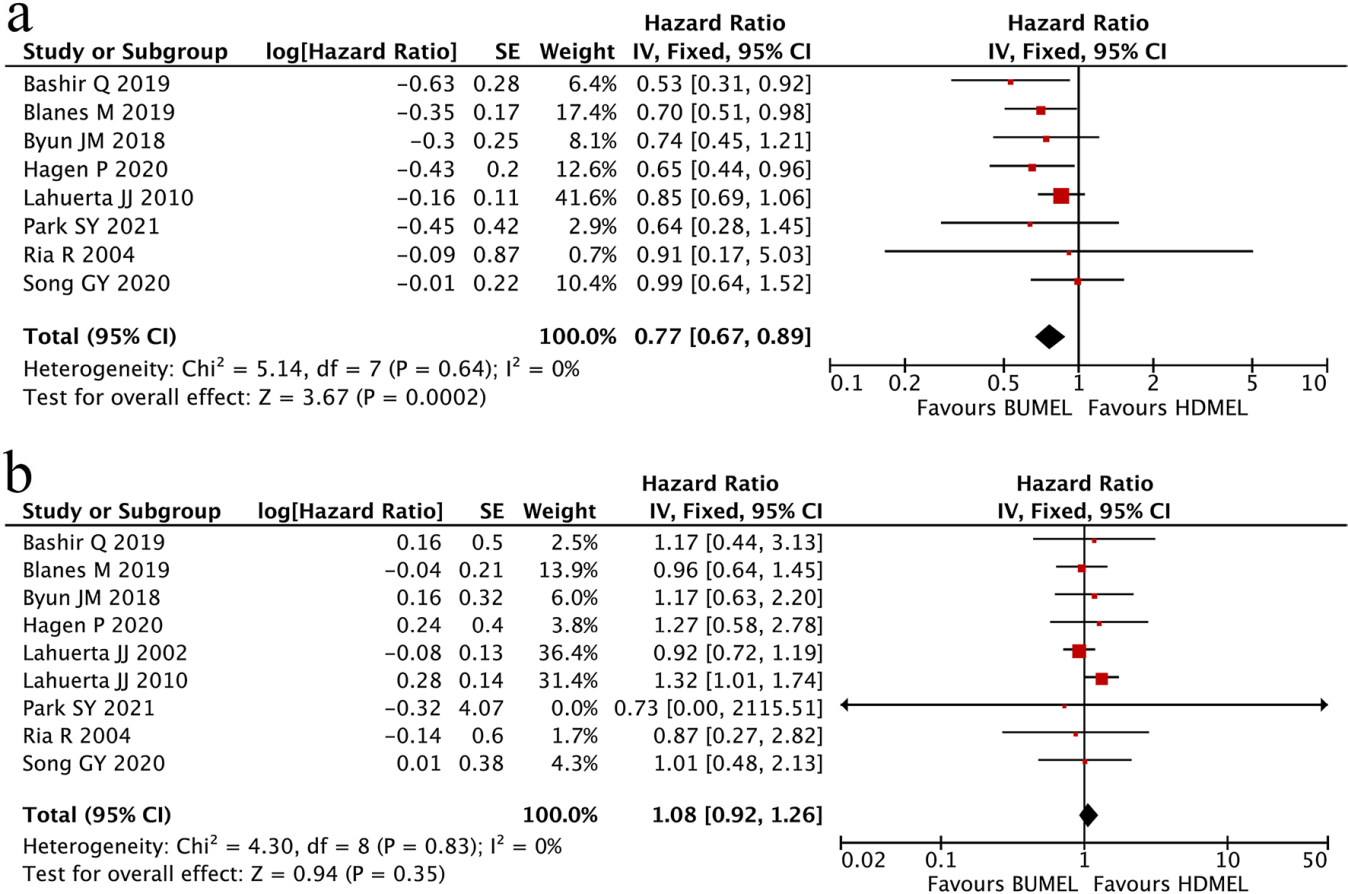
Comparisons of survivals between MM patients having undergone HDMEL and BUMEL-based conditioning regimens. **a** Progression-free survival (PFS); **b** overall survival (OS)

Among all 10 studies, 8 studies were available for analyses of PFS. The fixed-effects model was used to calculate the result as there was no heterogeneity among the included studies (I^2^ = 0%, P_heterogeneity_ = 0.64). Meta-analysis revealed that MM patients having received BUMEL-based conditioning regimen had significantly favorable PFS when compared with those having received HDMEL alone (HR 0.77; 95% CI 0.67~0.89, P = 0.0002; Fig. 2a).

A total of 9 studies were eligible for the assessment of OS. No significant difference was identified in OS between the two groups of patients (HR 1.08; 95% CI 0.92~1.26, P = 0.35; Fig. 2b) when using fixed-effects model to pool all of HRs and their 95% CIs (I^2^ = 0%, P_heterogeneity_ = 0.83).

### Best treatment response after auto-HSCT

**Fig. 3:**
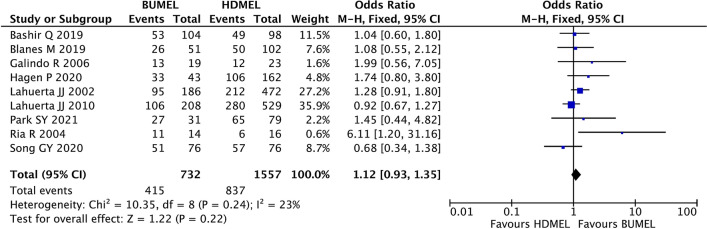
Comparisons of best treatment response after auto-HSCT between MM patients having undergone HDMEL and BUMEL-based conditioning regimens

The best treatment response after auto-HSCT was assessed in 9 studies. The fixed-effects model was applied to analyze this outcome due to no significant heterogeneity among the included studies (I^2^ = 23%, P_heterogeneity_ = 0.24). The result of meta-analysis indicated that no difference of the best response after auto-HSCT was found between patients in the BUMEL-based group and the HDMEL group (OR 1.12; 95% CI 0.93~1.35, P = 0.22; Fig. 3).

### Stem cell engraftment

**Fig. 4:**
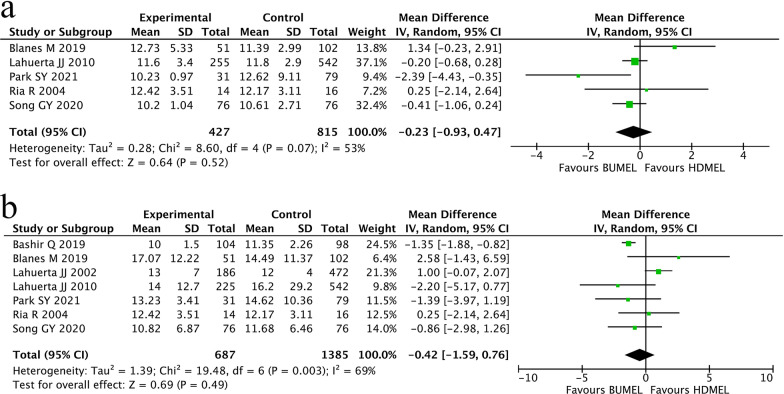
Comparisons of stem cell engraftment between MM patients having undergone HDMEL and BUMEL-based conditioning regimens. **a** Neutrophil engraftment; **b** platelet engraftment

Totally, 5 studies and 7 studies were eligible to assess neutrophil engraftment and platelet engraftment, respectively. Since obvious heterogeneity were observed among the mentioned studies, a random-effects model was employed to pool mean and SD. The combined results showed that there was no difference of the mean time to reach a neutrophil count ≥ 0.5*10^9^/L (MD − 0.23; 95% CI − 0.93~0.47, P = 0.52; Fig. 4a) or platelet count ≥ 20*10^9^/L (MD − 0.42; 95% CI − 1.59~0.76, P = 0.49; Fig. 4b) between the two groups of patients.

### Conditioning regimens-related toxicities

**Fig. 5:**
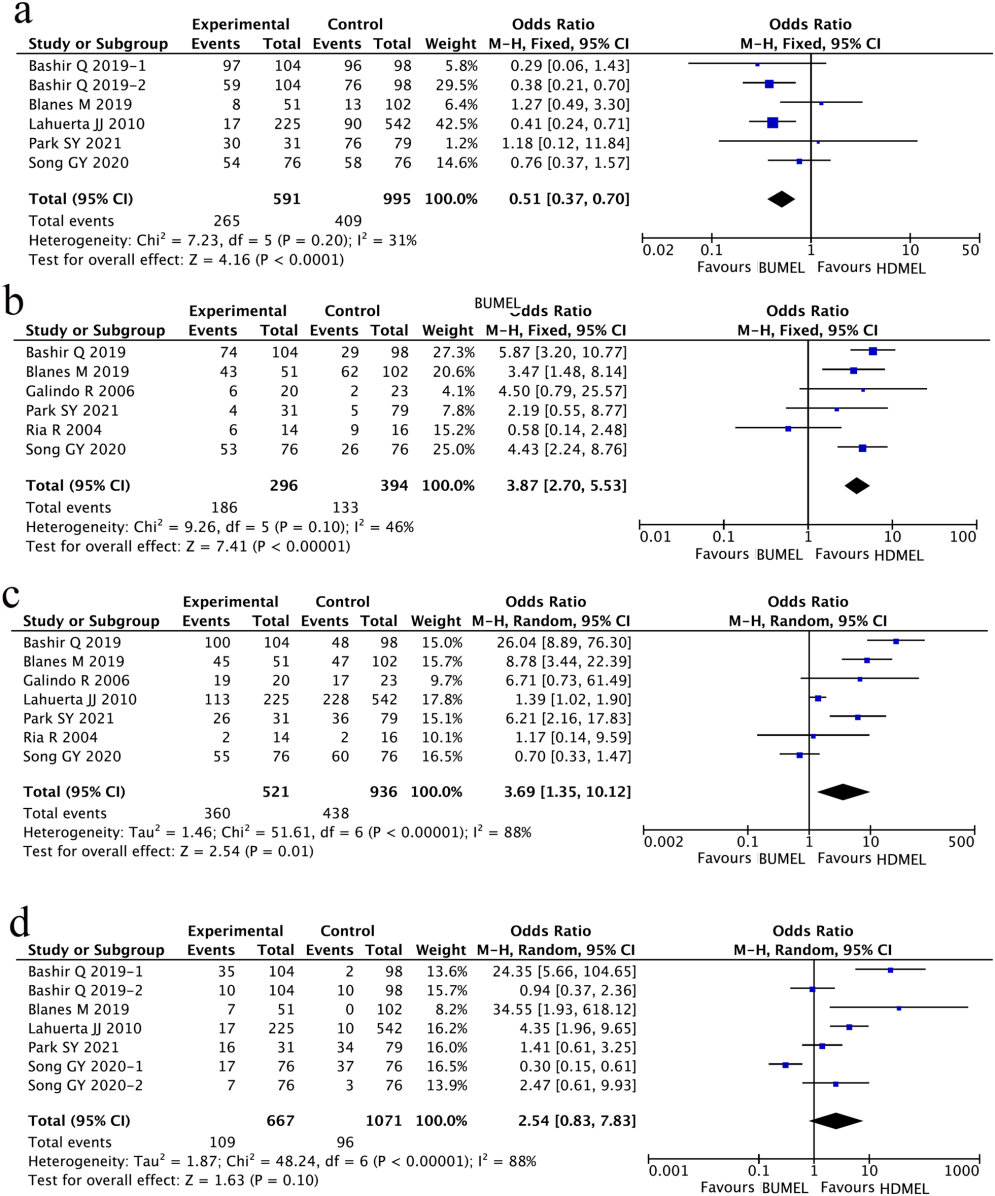
Comparisons of conditioning regimens-related toxicities between MM patients having undergone HDMEL and BUMEL-based conditioning regimens. **a** Gastrointestinal toxicity; **b** infection; **c** mucositis; **d** hepatic toxicity

The incidence of regimen-related toxicities (e.g., infection, mucositis, hepatic and gastrointestinal toxicity) were also comprehensively compared between patients in the BUMEL-based group and the HDMEL group. The results indicated that gastrointestinal toxicity was significantly less frequently observed in the BUMEL-based group than that in the HDMEL group (OR 0.51; 95% CI 0.37~0.70, P < 0.0001; Fig. 5a). However, the incidence of infection (OR 3.87; 95% CI 2.70~5.53, P < 0.00001; Fig. 5b) and mucositis (OR 3.69; 95% CI 1.35~10.12, P = 0.01; Fig. 5c) was significantly higher in patients having received BUMEL-based conditioning regimen compared with those having received HDMEL conditioning regimen. No significant difference was identified in the incidence of hepatic toxicity between the two groups of patients (OR 2.54; 95% CI 0.83~7.83, P = 0.10; Fig. 5d). Due to too low incidence of transplantation-related mortality in both groups of patients, we did not perform meta-analysis to compare transplantation-related mortality between the two groups.

### Sensitivity analysis

Sensitivity analysis was conducted by a sequential exclusion of individual studies to identify the origin of the heterogeneity and to verify the sensitivity of the results. The origin of the heterogeneity and overall effect after removal of origin of the heterogeneity for the respective outcome are listed in Table 2. As demonstrated from the results, the effect size for the respective outcome remained unchanged after the origin of the heterogeneity was removed, which demonstrated the stability of the meta-analysis results. However, for the outcomes of mucositis and hepatic toxicity, the heterogeneity remained even if studies were excluded one by one.

**Table 2:** Results of sensitivity analysis

Outcome	Origin of heterogeneity	Overall effect	P value
Neutrophil engraftment	Ria S 2004	− 0.25 (− 0.63~0.12)	0.18
Platelet engraftment	Bashir Q 2019	0.27 (− 0.62~1.06)	0.51

### Publication bias

Publication bias was assessed by Begg’s test and Egger’s test. The results were listed in Table 3. No significant publication bias was found for all the outcomes.

**Table 3:** Results of Begg’s test and Egger’s test

Outcome	P_Begg’s test_	P_Egger’s test_
Progression-free survival	0.902	0.376
Overall survival	0.917	0.990
Best treatment response	0.118	0.139
Neutrophil engraftment	1.000	0.994
Platelet engraftment	1.000	0.400
Infection	0.133	0.119
Mucositis	0.368	0.202
Hepatic toxicity	0.260	0.164
Gastrointestinal toxicity	0.452	0.462

## Discussion

Over the past two decades, considerable studies have investigated the impact of BUMEL conditioning regimen on auto-HSCT outcomes [12–15]. However, the results from the mentioned comparative studies remained elusive. Herein, the current study systematically compared the efficacy and toxicity of BUMEL-based conditioning with standard conditioning regimen HDMEL 200 mg/m^2^ in MM patients. There were several observations obtained.

As indicated from an existing study, improving pre-transplantation conditioning chemotherapy by applying higher doses of melphalan (220 mg/m^2^) in MM patients significantly increased toxicity (e.g., severe mucositis, delayed platelet engraftment, and cardiac arrhythmia), without clear survival advantage over melphalan at 200 mg/m^2^ despite the improvement of treatment response [10]. Herein, other groups attempted to improve auto-HSCT outcome via combining busulfan with other conditioning regimens, such as melphalan, cyclophosphamide, thiotepa, etoposide, etc. to improve auto-HSCT outcome [32–35]. Among them, BUMEL has been widely studied and found to be correlated with some benefit compared with HDMEL 200 mg/m^2^. In the current study, the most noticeable finding was the longer PFS found in MM patients having received conditioning of BUMEL-based as compared with those having received HDMEL 200 mg/m^2^, which might be explained by the synergistic effect of the combination of busulfan and melphalan [15]. Bashir Q, et al. observed the synergistic toxicity and activation of apoptosis when MM.1R and H929 MM cells were exposed to busulfan plus melphalan combination. The exact mechanism of synergism of these two alkylating agents remains unclear. Researchers speculated that the combination of busulfan with melphalan resulted in the formation of complex genomic lesions which were more difficult for the cancer stem cells to repair [15].

The favorable PFS identified in the BUMEL-based group did not translate into the advantage of OS. A possible interpretation referred to the subsequent lines of rescue treatment after relapse. Lahuerta JJ, et al. reported that the use of novel agents as salvage therapy showed an independent positive prognostic effect on OS regarding the use of conventional chemotherapy at relapse/progression [28]. Notably, in Lahuerta JJ’s study, patients underwent BUMEL conditioning between 2000 and 2002 while patients switched to HDMEL 200 mg/m^2^ conditioning after 2002 as impacted by the high incidence of veno-occlusive disease (VOD) [28]. This disparity in date resulted in less patients in BUMEL group access to novel agents as salvage treatments than those in HDMEL 200 mg/m^2^ group, which might have a critical impact on OS. Additionally, the different percentage of patients having received maintenance treatment and different maintenance treatment regimens between the two groups of patients might also have significant influence on OS.

In terms of toxicity, we observed that the incidence of gastrointestinal events was significantly lower in the BUMEL-based group as compared with that in the HDMEL group, which might be explained by the reduced dose of melphalan in the BUMEL-based group as gastrointestinal toxicity, such as diarrhea, nausea, and vomiting, was one of the most common adverse effects exerted by melphalan. However, infection and mucositis were more frequently identified among BUMEL-based recipients for higher intensity of the combined alkylating agent conditioning regimen of busulfan and melphalan. Nevertheless, the mentioned adverse events were manageable and did not extend to transplant-related mortality. Besides, the application of intravenous busulfan with pharmacokinetic dose adjustment could be conducive to reducing the incidence of VOD and other hepatic toxicity. Thus, the frequency of hepatic event was similar between the two conditioning groups in the existing study. Given the toxicity of melphalan and busulfan, delivery melphalan and busulfan simultaneously or separately by polymer nanoparticles may be a promising pharmaceutical design in clinic to reduce the adverse effect and further improve the efficacy of BUMEL conditioning regimen in the future [36].

To the best of the authors’ knowledge, this study is the first meta-analysis systematically assessing clinical outcomes between BUMEL-based and HDMEL as the conditioning regimen before auto-HCT for MM. We are confident that the results of this study were reliable, which was supported by large sample size, moderate to high methodological quality of included studies, low heterogeneity, and no publication bias. However, there were several limitations in this study. First, when evaluating OS or PFS, HR and 95% CI in some of individual studies were not available from the original studies, hence they were indirectly calculated from Kaplan-Meier curve. Second, minimal residual disease (MRD) testing has been widely used as a robust method for assessment of clinical efficacy of certain treatment regimen in MM [37, 38]. However, no individual studies in our meta-analysis included MRD as a primary clinical trial endpoint. Therefore, whether BUMEL-based conditioning could elevate MRD negativity rate compared with HDMEL conditioning remains unknown. Third, the heterogeneous characteristics of individual studies (e.g., its retrospective nature, small sample size, length of follow-up, historical controls, different induction, and post-transplantation treatments) might act as the confounding factors, thereby causing significant heterogeneity among included studies for several outcomes. Fourthly, although this study showed the superior PFS after auto-HSCT in patients receiving BUMEL-based conditioning regimen, unfortunately we still did not know best treatment response before auto-HSCT and the optional treatment after auto-HSCT. Last but not least, after performing comprehensive literature search, we found that nine of ten included studies were retrospective studies. Due to the retrospective nature, the evidence of the individual study was insufficient.

## Conclusions

The present meta-analysis revealed the high anti-myeloma activity of BUMEL-based conditioning prior to auto-HSCT in MM, which could be correlated with superior PFS and comparable OS compared with HDMEL conditioning though a higher incidence of manageable adverse events was found. BUMEL-based conditioning regimen is likely to act as an appropriate strategy to further improve long-tern outcomes in MM patients. Meanwhile, we should also pay attention to toxicity of BUMEL-based conditioning regimen and appropriate management is required. In view of the existing limitations of the current study, a well-designed prospective study with large cohorts should be conducted to clarify the mentioned issues in the future.

## Supplementary Information

**Additional file 1: Table S1.** PRISMA 2020 Checklist. **Table S2.** The details of the search strategy. 

**Additional file 2: Figure S1.** MINORS Scale to assess the study quality.

## Data Availability

The datasets used in this study are available from the corresponding author upon reasonable request.

## Abbreviations

auto-HSCT: Autologous hematopoietic stem cell transplantation
BUMEL: Busulfan plus melphalan
CI: Confidence interval
HDMEL: High-dose melphalan
HR: Hazard ratio
IMiDs: Immunomodulatory drugs
MD: Mean difference
MINORS: Non-Randomized Studies
MM: Multiple myeloma
MRD: Minimal residual disease
OS: Overall survival
PFS: Progression-free survival
PIs: Proteasome inhibitors
PRISMA: Preferred Reporting Items for Systematic Reviews and Meta-analysis
RCT: Randomized controlled trial
SD: Standard deviation
VGPR: Very good partial response
VOD: Veno-occlusive disease
