# Supplementary material for: Long-term outcomes of busulfan plus melphalan-based versus melphalan 200 mg/m2 conditioning regimens for autologous hematopoietic stem cell transplantation in patients with multiple myeloma: a systematic review and meta-analysis
Source: Cancer Cell Int. 2021 Nov 10;21:601. doi: 10.1186/s12935-021-02313-z (PMC8579671; doi:10.1186/s12935-021-02313-z)
Supplement: Supplementary file 2 — Additional file 2: Figure S1. MINORS Scale to assess the study quality. [file 12935_2021_2313_MOESM2_ESM.pdf]

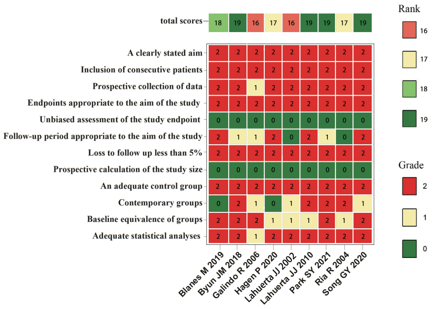
total scores
18 19 16 17 16 19 19 17 19
A clearly stated aim
2 2 2 2 2 2 2 2 2
Inclusion of consecutive patients
2 2 2 2 2 2 2 2 2
Prospective collection of data
2 2 1 2 2 2 2 2 2
Endpoints appropriate to the aim of the study
2 2 2 2 2 2 2 2 2
Unbiased assessment of the study endpoint
0 0 0 0 0 0 0 0 0
Follow-up period appropriate to the aim of the study
2 1 1 2 0 2 1 0 2
Loss to follow up less than 5%
2 2 2 2 2 2 2 2 2
Prospective calculation of the study size
0 0 0 0 0 0 0 0 0
An adequate control group
2 2 2 2 2 2 2 2 2
Contemporary groups
0 2 1 0 1 2 2 2 1
Baseline equivalence of groups
2 2 2 1 1 1 2 1 2
Adequate statistical analyses
2 2 1 2 2 2 2 2 2
Blanes M 2019
Byun JM 2018
Galindo R 2006
Hagen P 2020
Lahuerta JJ 2002
Lahuerta JJ 2010
Park SY 2021
Ria R 2004
Song GY 2020
Rank
16
17
18
19
Grade
2
1
0
